# Exploring How Dopants Strengthen Metal-Ni/Ceramic-Al_2_O_3_ Interface Structures at the Atomic and Electronic Levels

**DOI:** 10.3390/molecules30091990

**Published:** 2025-04-29

**Authors:** Fengqiao Sun, Xiaofeng Zhang, Long Li, Qicheng Chen, Dehao Kong, Haifeng Yang, Renwei Li

**Affiliations:** 1 Jilin Institute of Chemical Technology, College of Aeronautical Engineering, Jilin 132022, China; 2 Public Education Department, Gongqing Institute of Science and Technology, Gongqing 332020, China; 3 School of Energy and Power Engineering, Northeast Electric Power University, Jilin 132012, China; 4 College of New Energy and Materials, Northeast Petroleum University, Daqing 163711, China; 5 State Key Laboratory of Advanced Welding and Joining, Harbin Institute of Technology, Weihai 264209, China; 6 Jilin Institute of Chemical Technology, College of Mechanical and Electrical Engineering, Jilin 132022, China

**Keywords:** metal–ceramic interface, electronic properties, strengthening mechanism

## Abstract

The metal-based/ceramic interface structure is a key research focus in science, and addressing the stability of the interface has significant scientific importance as well as economic value. In this project, the work of adhesion, heat of segregation, electronic structure, charge density, and density of states for doped-M (M = Ti, Mg, Cu, Zn, Si, Mn, or Al) Ni (111)/Al_2_O_3_ (0001) interface structures are studied using first-principle calculation methods. The calculation results demonstrate that doping Ti and Mg can increase the bonding strength of the Ni–Al_2_O_3_ interface by factors of 3.4 and 1.5, respectively. However, other dopants, such as Si, Mn, and Al, have a negative effect on the bonding of the Ni–Al_2_O_3_ interface. As a result, the alloying elements may be beneficial to the bonding of the Ni–Al_2_O_3_ interface, but they may also play an opposite role. Moreover, the Ti and Mg dopants segregate from the matrix and move to the middle position of the Ni–Al_2_O_3_ interface during relaxation, while other dopants exhibit a slight segregation and solid solution in the matrix. Most remarkably, the segregation behavior of Ti and Mg induced electron transfer to the middle of the interface, thereby increasing the charge density of the Ni–Al_2_O_3_ interface. For the optimal doped-Ti Ni–Al_2_O_3_ interface, bonds of Ti–O and Ti–Ni are found, which indicates that the dopant Ti generates stable compounds in the interface region, acting as a stabilizer for the interface. Consequently, selecting Ti as an additive in the fabrication of metal-based ceramic Ni–Al_2_O_3_ composites will contribute to prolonging the service lifetime of the composite.

## 1. Introduction

Metal-based ceramic composites are advanced, interesting materials whose performances are based on a combination of metal and ceramics. They are widely used in industrial fields, such as aviation, energy, sensors, and automotive [1,2,3,4]. Metal-based ceramic composites mainly include a metal–ceramic coating and metal-based-reinforced ceramic particles. It is worth noting that as a novel Ni–Al_2_O_3_ composite, the interface between Ni and Al_2_O_3_ determines the stability and lifespan of the composite. Therefore, it is of great scientific significance to explore the properties of the Ni–Al_2_O_3_ interface.

**Figure 1:**
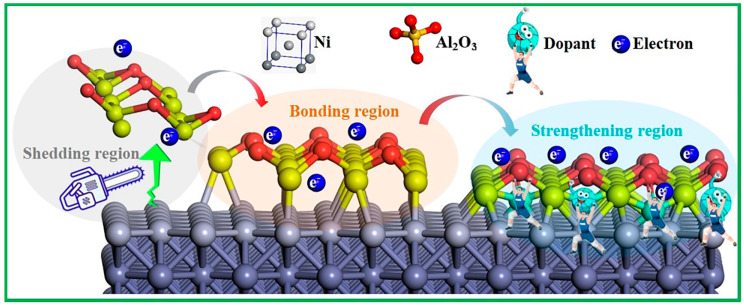
An overview of the Ni–Al_2_O_3_ interface region.

Regarding experiments, most studies primarily deal with the production process of metal-based ceramic composites and assess their chemical stability and mechanical characteristics. For instance, research conducted by Martínez-Franco et al. [5] found that a Ni–Al_2_O_3_ composite prepared through the high-kinetic processing of ball milling and spark plasma sintering possessed excellent mechanical properties, making it a potential candidate for various high-temperature applications. Just recently, Gao et al. [6] showed that ceramic nanoparticle-enhanced multilayer Ni–Ni–Al_2_O_3_ coating structures exhibited superior toughness, wear resistance, and corrosion resistance. Moreover, it was discovered that this multilayer coating structure was less prone to cracking. Shen et al. [7] suggested that an Ni–Al_2_O_3_ coating pretreatment is expected to develop as a low-cost, novel, and effective method for promoting nitride. However, Fu et al. [8] suggested that the Ni–Al_2_O_3_ interface can crack and expand under hot conditions so that the material could fail early. Our early experiments [9] have also confirmed this. Currently, the biggest challenges faced by Ni–Al_2_O_3_ composites during manufacturing and use are the following: (1) poor wettability between the matrix and ceramic; and (2) weak bonding strength at the matrix–ceramic interface. Indeed, effective dopants would be an effective measure to strengthen the Ni–Al_2_O_3_ interface binding, as shown in Figure 1. Therefore, it is necessary to conduct a more in-depth exploration of the modification of the Ni–Al_2_O_3_ interface, especially at the atomic and electronic scales.

First-principle calculations based on quantum mechanics can study the properties of metal–ceramic interfaces, shedding light on the microscopic features at minimal material dimensions. Hocker et al. [10] applied first-principles calculations to predict the tensile properties of metal–ceramic interfaces at the electronic dimension. Our team [11,12] found that active alloying elements and rare earth elements can act as binders, reducing interfacial spacing and enhancing the bonding strength of the Fe–Al_2_O_3_ interface. Research by Chen et al. [13] has shown that doping Mg and Cu can make the charge distribution on the Al–Al_2_O_3_ interface uniform, thereby enhancing its mechanical properties. Chen et al. [14] successfully predicted the fracture process of the NiTi–Al_2_O_3_ interface, thereby obtaining the failure mechanism of the structures. Guo et al. [15] found that dopant-Ti can form strong Ti–O ionic bonds at the Ag–Al_2_O_3_ interface, enhancing the bonding strength of the interface. Consequently, it can be seen that employing the first-principles calculation method can obtain the properties of the interface and reveal the bonding mechanism of heterogeneous materials from the atomic and electronic scales, a task that is unachievable through experimental methods.

Only very recently, our group has conducted research on the properties of Ni–Al_2_O_3_ interfaces and discovered that there are three types of Ni–Al_2_O_3_ interfaces (single Al-terminated, double Al-terminated, and O-terminated), which have significant differences in their bonding strengths [16]. Among them, the single Al-terminated Ni–Al_2_O_3_ interface is the easiest to form in the actual environment, serving as the basic model for this research. This work primarily involves doping alloying elements into the single Al-terminated Ni–Al_2_O_3_ interface, aiming to optimize the interfacial environment and strengthen the interfacial bonding. The research details are presented below. Firstly, we utilized the work of adhesion to screen for dopants-M (M = Ti, Mg, Cu, Zn, Si, Mn, or Al), which are advantageous for the bonding of the Ni–Al_2_O_3_ interface. Following this, we undertook a detailed atomic and electronic structural analysis of the Ni–Al_2_O_3_ interfaces showing positive effects, thereby uncovering their micro-level characteristics. In the final stage, we conducted comprehensive testing on the best-performing doped-Ti Ni–Al_2_O_3_ interface conditions with the aim of elucidating the specific mechanisms behind the strengthening effects at this interface.

## 2. Results and Discussion

### 2.1. The Binding Strength of the Interface

Generally, the work of adhesion (abbreviated here as the *W_ad_*) can be used to evaluate the binding strength of the interface semi-quantitatively, as one of the criteria for judging the bonding properties of heterogeneous materials. In this work, the *W_ad_* of the doped-M (M = Ti, Mg, Cu, Zn, Si, Mn, or Al) and non-doped Ni–Al_2_O_3_ interface structures are calculated to reveal the effect of doping on the bonding of the interface, as shown in the following Equation (1) [17,18]:

(1) $$W_{ad}=\frac{E_{Ni+M}+E_{Al_{2}O_{3}}-E_{Ni+M/Al_{2}O_{3}}}{A}$$

Here, *E_Ni+M_* and $E_{{Al}_{2}O_{3}}$ denote the total energies of the Ni-side with dopant-M and Al_2_O_3_-side, respectively. $E_{Ni+M/{Al}_{2}O_{3}}$ is the total energy of the doped-M Ni–Al_2_O_3_ interface structure, and A stands for the area of the Ni–Al_2_O_3_ interface.

**Figure 2:**
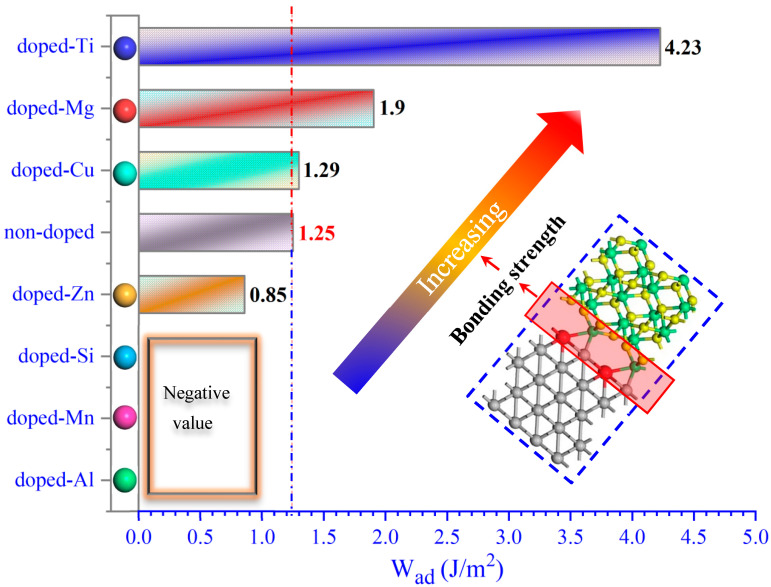
The work of adhesion (W_ad_) of non-doped and doped-elements in Ni–Al_2_O_3_ interface.

Figure 2 shows the *W_ad_* of the doped-M and non-doped Ni–Al_2_O_3_ interface structures. In contrast to the non-doped Ni–Al_2_O_3_ interface, the *W_ad_* of the doped-M Ni–Al_2_O_3_ interfaces, in descending order, is the doped-Ti (4.23 J/m^2^) > doped-Mg (1.9 J/m^2^) > doped-Cu (1.29 J/m^2^) > non-doped (1.25 J/m^2^) > doped-Zn (0.85 J/m^2^), showing that Ti and Mg has a positive effect on strengthening the bonding of the Ni–Al_2_O_3_ interface. Yet, the Zn dopant weakens the bonding strength between the Ni and Al_2_O_3_. More noteworthy, the *W_ad_* of the Ni–Al_2_O_3_ interfaces doped with Si, Mn, and Al are negative, suggesting that these dopants would even prevent the formation of the interface. The main reasons for this phenomenon include the compatibility between elements in materials and the activity of the atoms (energy and size, etc.). In a previous study by our group [19], it was also found that titanium can improve the tensile properties of the Fe–Al_2_O_3_ interface structure. In this way, Ti seems to be effective in improving the interface properties of metals and Al_2_O_3_, which requires further verification with similar metals–Al_2_O_3_ structures. Consequently, doping alloy elements in the Ni–Al_2_O_3_ interface can have an uncertain effect on the bonding of that interface, which may be enhanced or weakened.

The process of doping will cause changes in the diameter and energy of the atomic position, which will induce a relaxation behavior in the atoms to form a new interface structure. The heat of segregation (Δ*G_seg_*) is the driving force that forms the stable compound, which can be used to evaluate the stability of dopants in the interface structure. The calculation of Δ*G_seg_* is performed utilizing the following Equation (2) [20]:

(2) $$\Delta G_{seg}=\frac{1}{n}\left(E_{Ni/Al_{2}O_{3}}-E_{Ni/Al_{2}O_{3}.nx}+n\mu_{x}-n\mu_{Ni}\right)$$

where $E_{Ni/{Al}_{2}O_{3}}$ and $E_{Ni/{Al}_{2}O_{3}.nx}$ represent the total energies of the non-doped and doped-M Ni–Al_2_O_3_ interface structures, respectively. Here, *n* denotes the total number of atoms doped with *x* (where *x* can be Ti, Mg, Cu, Zn, Si, Mn, or Al), and *μ* signifies the chemical potential of the dopant element. If Δ*G_seg_* is positive, it means that dopant atoms are easy to segregate from the matrix; if Δ*G_seg_* is negative, it indicates that the doped atoms are a solid solution in the matrix and not prone to segregation.

**Figure 3:**
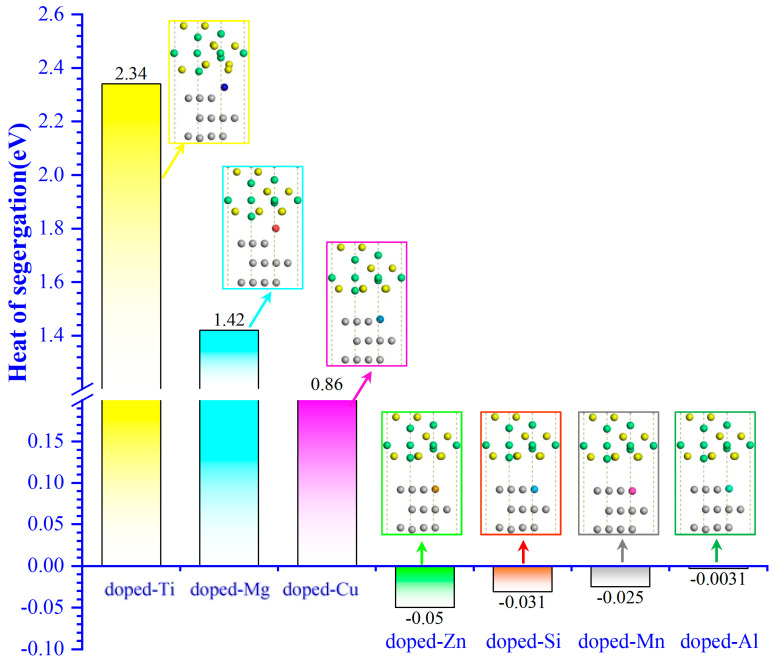
The heat of segregation (Δ*G*_seg_) for non-doped and doped Ni–Al_2_O_3_ interfaces.

Figure 3 shows the Δ*G_seg_* of the doped-M and non-doped Ni–Al_2_O_3_ interface structures. It is obvious that the Δ*G_seg_* of the doped-Ti, Mg, and Cu Ni–Al_2_O_3_ interface structures are larger than 0, which are 2.34, 1.42, and 0.86 eV, respectively, indicating that the dopants are in an unstable state in the matrix. However, the Δ*G_seg_* of the doped-Zn, Si, Mn, and Al Ni–Al_2_O_3_ interfaces are negative and very close to 0, which means that the dopants are more prone to solid solutions in the matrix. From the perspective of the atomic structure, the Ti and Mg atoms have a large relaxation behavior, and the remaining dopants change very little, which matches the result of the Δ*G_seg_*. Indeed, a higher heat of segregation corresponds to a lower total energy in the atomic structure’s system, resulting in a more stable structure. Taken together, Ti, Mg, and Cu dopants are beneficial to the stability of the Ni–Al_2_O_3_ interface, and hence they are selected for further studies.

### 2.2. The Electronic Properties of the Interface

The formation of heterogeneous materials is complex, especially with chemical and physical changes at the interface region, which will induce a transfer and redistribution of electrons. To better understand the strengthening mechanism of the Ni–Al_2_O_3_ interface by doping elements, it is necessary to study the electronic properties of the interfacial region. Next, we will discuss the spatial distribution of the electrons, the charge density of the plane, the electron overlap population, and the density of states for the Ni–Al_2_O_3_ interface structures.

**Figure 4:**
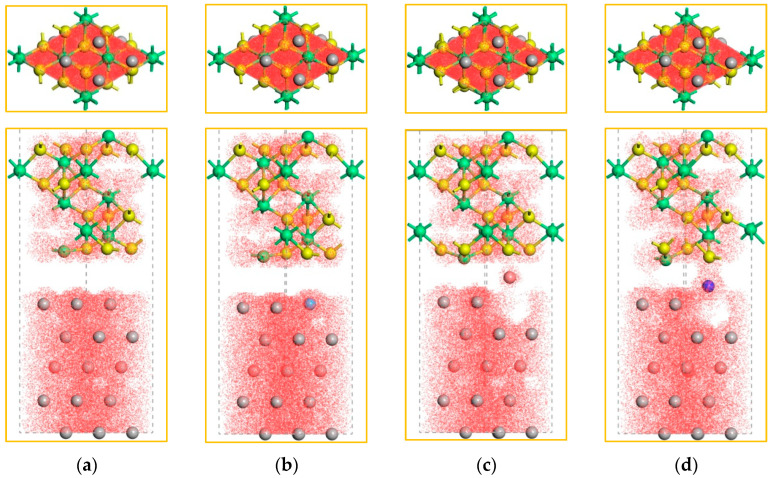
The electronic distribution state of non-doped and doped Ni–Al_2_O_3_ interfaces. (**a**) non-doped. (**b**) doped-Cu. (**c**) doped-Mg. (**d**) doped-Ti.

Figure 4 shows the spatial electron distribution of the non-doped and doped-M (M = Ti, Mg, and Cu) Ni–Al_2_O_3_ interface structures. In general, the electron distribution positions mean the interactions between the atoms. Compared to the non-doped Ni–Al_2_O_3_ interface, Mg and Ti dopants relax towards the center of the interface, transferring their surrounding electrons onto the interface. Interestingly, it is the segregation of the dopant that allows the electrons to be continuously distributed to connect the metal and the ceramic (see Figure 4c,d), which is a microscopic manifestation of the strengthening effect. It is worth noting that the number of electrons at the doped-Ti interface is more than that of other interfaces, indicating that Ti has the most significant effect on the Ni–Al_2_O_3_ interface. This phenomenon matches the results of the *W_ad_* above. Moreover, we found that Cu had no obvious effects on the electronic environment of the Ni–Al_2_O_3_ interface. Consequently, effective dopants can modify the interfacial electronic environment, thereby achieving the purpose of the strengthening effect. To put it differently, the properties of the interface are dictated by the electronic environment.

**Figure 5:**
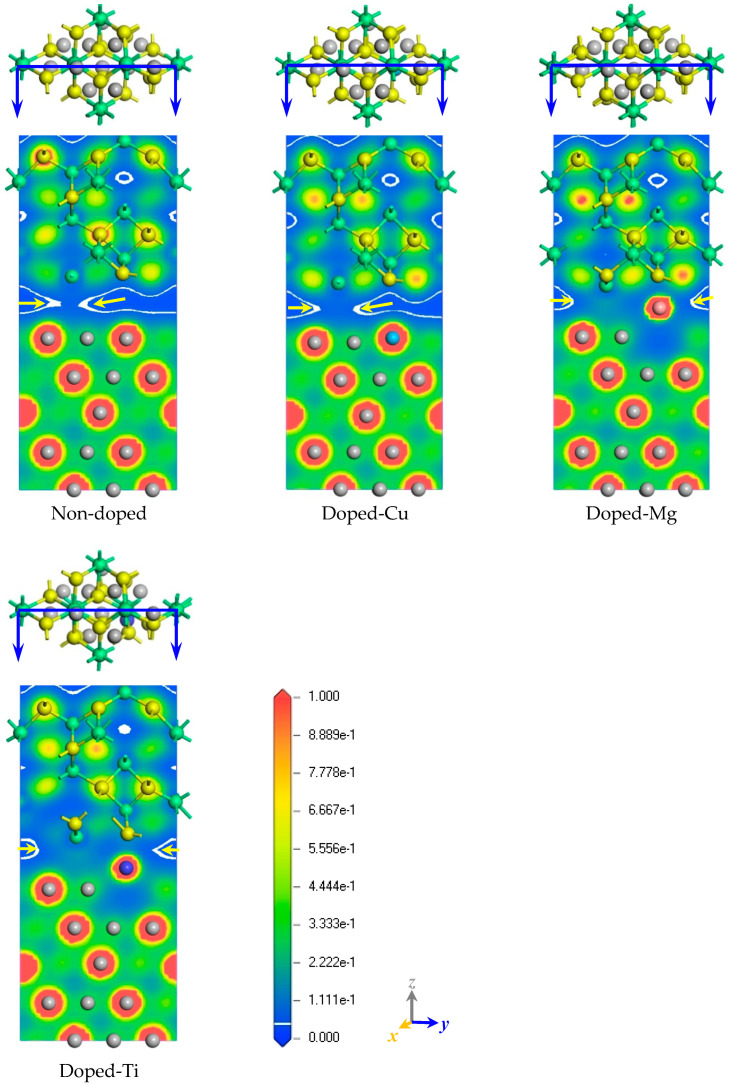
The charge density of non-doped and doped Ni–Al_2_O_3_ interfaces.

In order to better understand the characteristics of the Ni–Al_2_O_3_ interface, the charge density of the plane was analyzed, as shown in Figure 5. The plane with the greatest number of atoms is presented in this work, where red represents the charge aggregation region and blue represents the charge depletion region.

The distribution of charge density on the Ni-side and Al_2_O_3_-side are regular, indicating that the doping behavior of the interfacial region did not affect the non-interfacial region. The introduction of dopants (Ti, Mg, or Cu) at the Ni–Al_2_O_3_ interface widens the charge distribution region relative to the non-doped interface (illustrated by the yellow arrow in the Figure 5), marking a stronger bonding interaction from the interface. In particular, the Mg and Ti atoms had relaxed to the middle of the interface, effectively increasing the charge density of the interface. Herein, the segregation behavior of the dopants induces a lattice distortion at the interface, which may be beneficial for the bonding of the Ni–Al_2_O_3_ interface. Among all the doped Ni–Al_2_O_3_ interfaces, the reinforcement effect of Ti on interfacial bonding was the most significant, making it the best choice for practical applications. Therefore, further research on the strengthening mechanism of doped-Ti Ni–Al_2_O_3_ interface is necessary.

The overlap population of bonds provides a direct means to assess whether a valid chemical bond forms between atoms and unveils insights into the chemical changes occurring in the interfacial region. According to the traditional Mulliken formula (Equation (3)) [21], the overlap population of electrons for chemical bonds (Z*_bond_*) can be derived by using Equation (4). Both of these equations are expressible as the following:

(3) $$X_{Mulliken}=\frac{A+I}{2}$$

(4) $$Z_{bond}=ae^{-b(\Delta X_{Mulliken}{)}^{2}}+c$$

Here, *A* represents the affinity energy of the atom, *I* represents the ionization energy of the atom, and a, b, and c are constants. The *Z_bond_* represents the electron overlap population for the bonds, with a greater value indicating a greater strength of bond. A Z*_bond_* value greater than 0 indicates that the two atoms are in a bonded state, while a Z*_bond_* value less than 0 indicates that the two atoms are in a non-bonded state.

**Figure 6:**
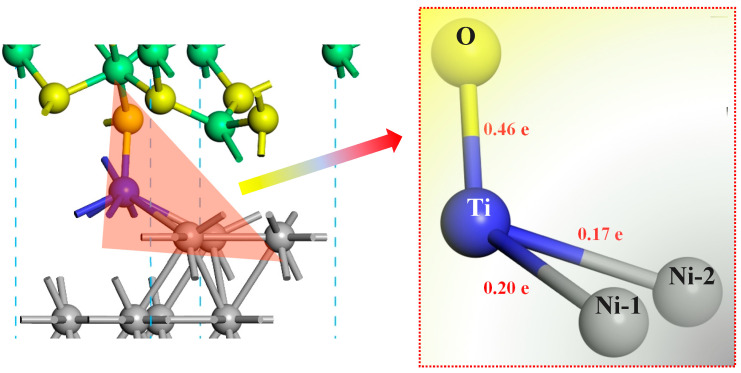
The overlap population of bonds for the doped-Ti Ni–Al_2_O_3_ interface.

Figure 6 is the overlap population of bonds at the doped-Ti Ni–Al_2_O_3_ interface. The values of overlap populations between Ti and O, between Ti and Ni-1, and between Ti and Ni-2 at the interface were 0.46, 0.2, and 0.17 e, respectively. This demonstrates that Ti segregates to the interface and undergoes chemical reactions, forming new chemical bonds with the atoms at both sides of the interface. In fact, the formation of new chemical bonds progressively integrates the two interfacial sides into a unified entity. Therefore, it can be inferred that the Ti undergoes complex chemical reactions in the Ni–Al_2_O_3_ interface region and produces new substances that contribute to the interface’s stability, which is the strengthening mechanism of the Ni–Al_2_O_3_ interface. As a result, the dopant acts as an adhesive at the interface, which can be understand as a “glue effect”.

**Figure 7:**
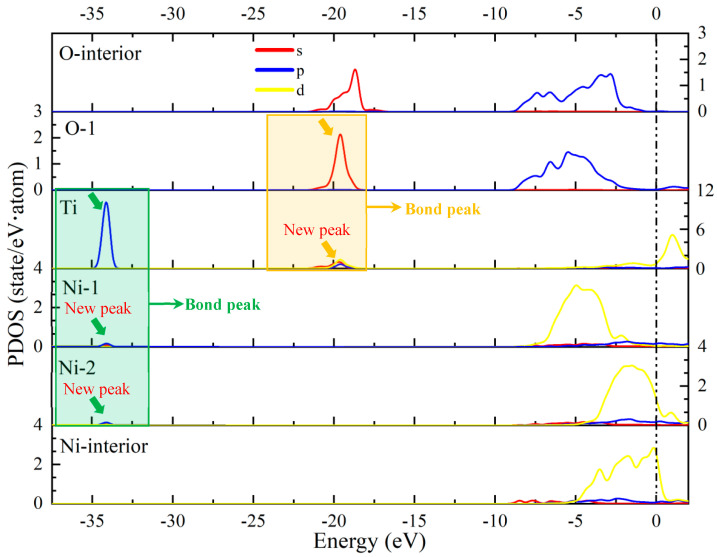
The PDOS of doped-Ti Ni–Al_2_O_3_ interface. (The position of the atoms is shown in Figure 6).

In order to reveal the bonding mechanism at the doped-Ti Ni–Al_2_O_3_ interface, we calculated the partial density of states (PDOSs) of the atoms. Figure 7 shows the PDOS of the doped-Ti Ni–Al_2_O_3_ interface (the positions of the atoms are provided in Figure 6). Relative to the Ni-interior atom, the electron *p*-orbits of the Ni-1 and Ni-2 at the interface exhibit new peaks ranging from −34.57 to −33.58 eV, matching the shape of the *p*-orbit of the Ti atom. It can be suggested that the orbital hybridization of electrons has occurred between the Ti and Ni-1, as well as between the Ti and Ni-2, which are a characteristic feature of covalent bonds. Furthermore, the peak of the s-orbit of O-1 atom at the interface becomes stronger within the range of −21.34 to −18.25 eV. The *s*, *p*, and *d*-orbits of the Ti atoms exhibited peaks that match the shape of the *s*-orbit of the O-1 atom. It can be concluded that electrons are transferred from Ti to O-1, and orbital hybridization of electrons occurs between the Ti and O-1, resulting in the formation of a Ti–O ionic compound. Consequently, the Ti–O1, Ti–Ni1, and Ti–Ni2 bonds exhibit typical bonding peaks (see the green and yellow boxes in Figure 7). This bonding behavior promotes and accelerates the formation of a stable Ni–Al_2_O_3_ interface.

Here, our conclusions will provide theoretical guidance for practical applications, advancing the innovation of Ni–Al_2_O_3_ composites. In future work, our group will consider the effect of the defects of the materials on the Ni–Al_2_O_3_ interface and the strengthening effect of the dopants on the interface under the defects.

## 3. Calculation Method and Details

### 3.1. Calculation Parameter

For this research, every calculation, from bulks to surfaces and interfaces, are executed with the Cambridge Sequential Total Energy Package (CASTEP) code [22], which is based on density functional theory (DFT). Initially, the geometry optimization of unit cells, surfaces, and interfaces are carried out using the Broyden–Fletcher–Goldfarb–Shanno (BFGS) minimization algorithm [23]. Convergence tests confirmed that a plane-wave cutoff energy of 380 eV is sufficient. The Brillouin zone was sampled using an 8 × 8 × 1 k-point grid for both interface and surface calculations. To ensure the accuracy of the calculations, the optimization convergence criteria for the original crystal cell structure was set as the following: an energy deviation per atom of 0.01 meV, a stress deviation of 0.05 GPa, and a maximum displacement of 0.001 Å. Subsequently, the ultrasoft pseudopotential (USPP) [24] was used to describe the interaction between the ionic nucleus and valence electrons. The valence electrons of the three atoms are set as O-2s^2^2p^4^, Al-3s^2^3p^1^, and Ni-3d^8^4s^2^, respectively. Ultimately, the generalized gradient approximation (GGA) with the Perdew–Burke–Enzerh (PBE) [25] method served as the electron exchange and correlation function in this work.

### 3.2. Model Building

**Figure 8:**
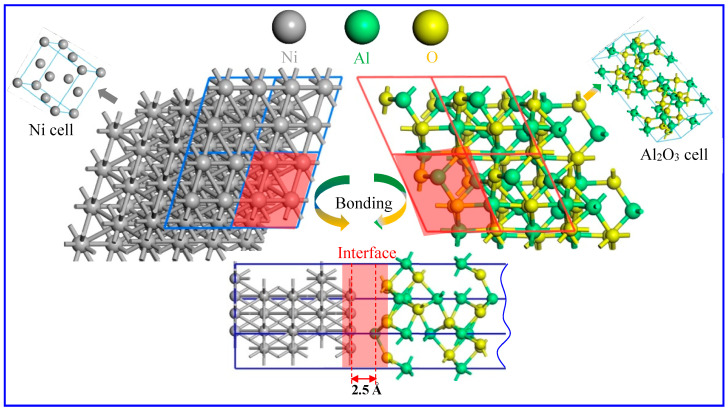
The schematic diagram of the Ni–Al_2_O_3_ interface formation.

According to the actual situation of material, the γ-Ni and α-Al_2_O_3_ cells serve as the basic units of the structure. The lattice constants for the Ni unit cell are a = b = c = 3.528 Å, and Al_2_O_3_ has a = b = 4.815 Å and c = 13.135 Å. Typically, stable heterogeneous interface systems are composed of crystal planes with the least energy of surface. It has been shown that γ-Ni (111) [17] and α-Al_2_O_3_ (0001) [26] belong to the surface structures with the lowest surface energy in the low-index crystal surfaces. Therefore, the α-Al_2_O_3_ (0001)/γ-Ni (111) interface system is established, which consists of 5 layers of Ni and 12 layers of Al_2_O_3_, as depicted in Figure 8.

**Figure 9:**
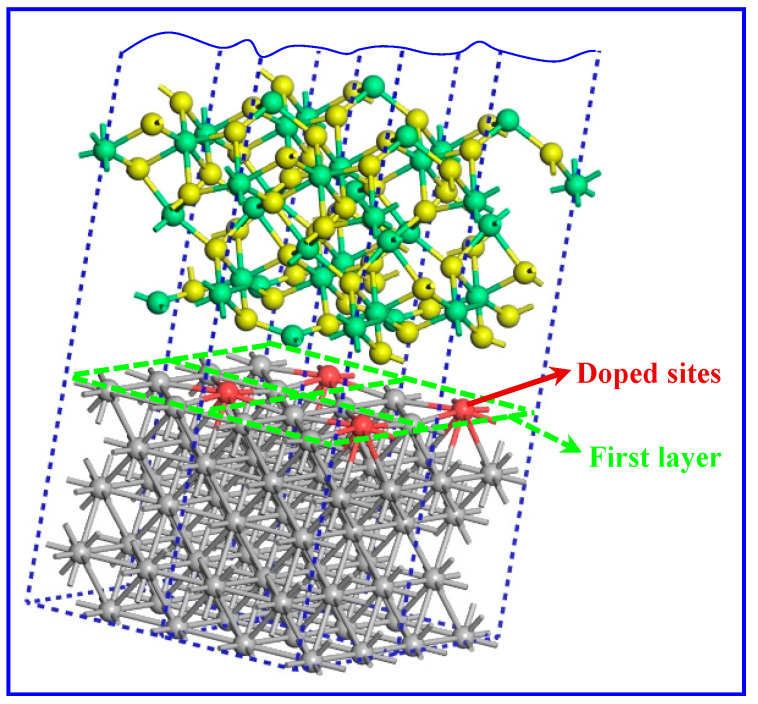
The supercell model of doped-*M* (*M* = Ti, Mg, Cu, Zn, Si, Mn, or Al) Ni–Al_2_O_3_ interface.

Furthermore, the outermost atomic layer on the surface of the material has the greatest influence on the bonding properties of the interface, so the central position of the interface layer was chosen as the doping site to achieve effective contrast, as shown in Figure 9.

## 4. Conclusions

In this work, we conducted a systematic investigation into the bonding strength and strengthening mechanism of Ni–Al_2_O_3_ interfaces with doped-M (M = Ti, Mg, Cu, Zn, Si, Mn, or Al) at the atomic and electronic scales, yielding the following conclusions:

(1) The doping of Ti and Mg can increase the work of adhesion of the Ni–Al_2_O_3_ interface by up to factors of 3.38 and 1.52, respectively, while the dopant Cu almost does not change the work of adhesion of the interface. Conversely, the dopants of Zn, Si, Mn, or Al can not only fail to improve the work of adhesion, but also weaken the bonding strength of the interface. Accordingly, different dopants may exert either positive or negative effects on interfacial bonding.

(2) The dopant Ti and Mg atoms will precipitate from the matrix Ni and diffuse into the middle of the Ni–Al_2_O_3_ interface in the relaxation process. More importantly, the diffusion behavior of the Ti and Mg can induce the transfer and redistribution of electrons in the Ni–Al_2_O_3_ interface region. Compared to the non-doped Ni–Al_2_O_3_ interface, the doping of Ti and Mg increase the number of electrons and broaden the area of charge distribution in the interface region. As a result, effective dopants can optimize the electronic structure and enhance interatomic bonding strength at the interface.

(3) Of all the doped Ni–Al_2_O_3_ interfaces, Ti is the best candidate. It is found that the stronger bonds of Ti–Ni and Ti–O are formed at the interface, which significantly improved the stability of the Ni–Al_2_O_3_ interface. In other words, the dopant Ti appears to act as an adhesive at the Ni–Al_2_O_3_ interface.

## Data Availability

The data presented in this study are available in article.
